# Recurrent Spontaneous Pneumothorax Associated With Marijuana Abuse: Case Report and Literature Review

**DOI:** 10.7759/cureus.13205

**Published:** 2021-02-07

**Authors:** Nouraldeen Manasrah, Ali F Al Sbihi, Sarah Al Qasem, Rohan Naik, Malitha Hettiarachchi

**Affiliations:** 1 Internal Medicine, Detroit Medical Center (DMC) Sinai-Grace Hospital, Detroit, USA; 2 Emergency Department, Luzmila Hospital, Irbid, JOR; 3 Cardiology, University of Connecticut School of Medicine, Farmington, USA

**Keywords:** pneumothorax (ptx), marijuana abuse, emphysematous bullae

## Abstract

Marijuana is the most commonly used illicit recreational drug in the United States. Growing public support for marijuana law reform has resulted in a significant increase in its use. The harmful pulmonary consequences of chronic marijuana smoking are less researched and discussed than those of tobacco smoking. We present a case of recurrent spontaneous pneumothorax in a patient with heavy, persistent marijuana abuse who has no past medical or surgical histories and denied smoking cigarettes or other illicit substance use.

## Introduction

The prevalence of marijuana use in the United States has increased recently [1], especially as the laws and attitudes toward the use of marijuana are changing. This also gives rise to concerns regarding its potential risks to the body and brain. 

The medical benefits of marijuana have led its use in treating a variety of medical conditions, including pain, chemotherapy-induced nausea and vomiting, appetite loss, and high ocular pressure [2,3]. However, it has many side effects like altered senses, dizziness, fatigue, reduced coordination and balance, cognitive impairment, anxiety, paranoia, and hallucinations [4]. Lung damage caused by marijuana occurs over time, and short-term effects like coughing, wheezing, sputum production, and shortness of breath are well documented [5].

The harmful pulmonary consequences of chronic marijuana smoking are less well documented than those of tobacco smoking. Spontaneous pneumothorax has been described as a long-term consequence, but most reported cases are of patients who combined tobacco and cannabis, which makes it difficult to see the direct causational effects of long-term marijuana abuse on the lungs [6-9]. Our patient smokes marijuana heavily without tobacco, which gives us a chance to highlight its effect on the lungs and its potential to cause bullous lung disease leading to spontaneous pneumothorax.

## Case presentation

A 29-year-old male of average height and weight, with no past medical or surgical conditions, presented to the emergency room with complaints of multiple episodes of vomiting followed by the sudden onset of right-sided chest pain and shortness of breath. The chest pain was severe and sharp in nature and increased with deep breathing. He reported smoking seven joints of marijuana every day for the past ten years and denied smoking cigarettes or other illicit substance use. Vital signs on admissions were significant for tachypnea (36 breaths/min), tachycardia (108 beats/min), with a blood pressure of 138/85 mmHg. Cardiovascular examination revealed tachycardia with regular rhythm, normal S1 and S2, with no murmurs. The respiratory examination revealed hyper-resonance on percussion with decreased breath sounds over the right side of the chest, suggesting pneumothorax. The rest of the physical examination was unremarkable.

**Figure 1:**
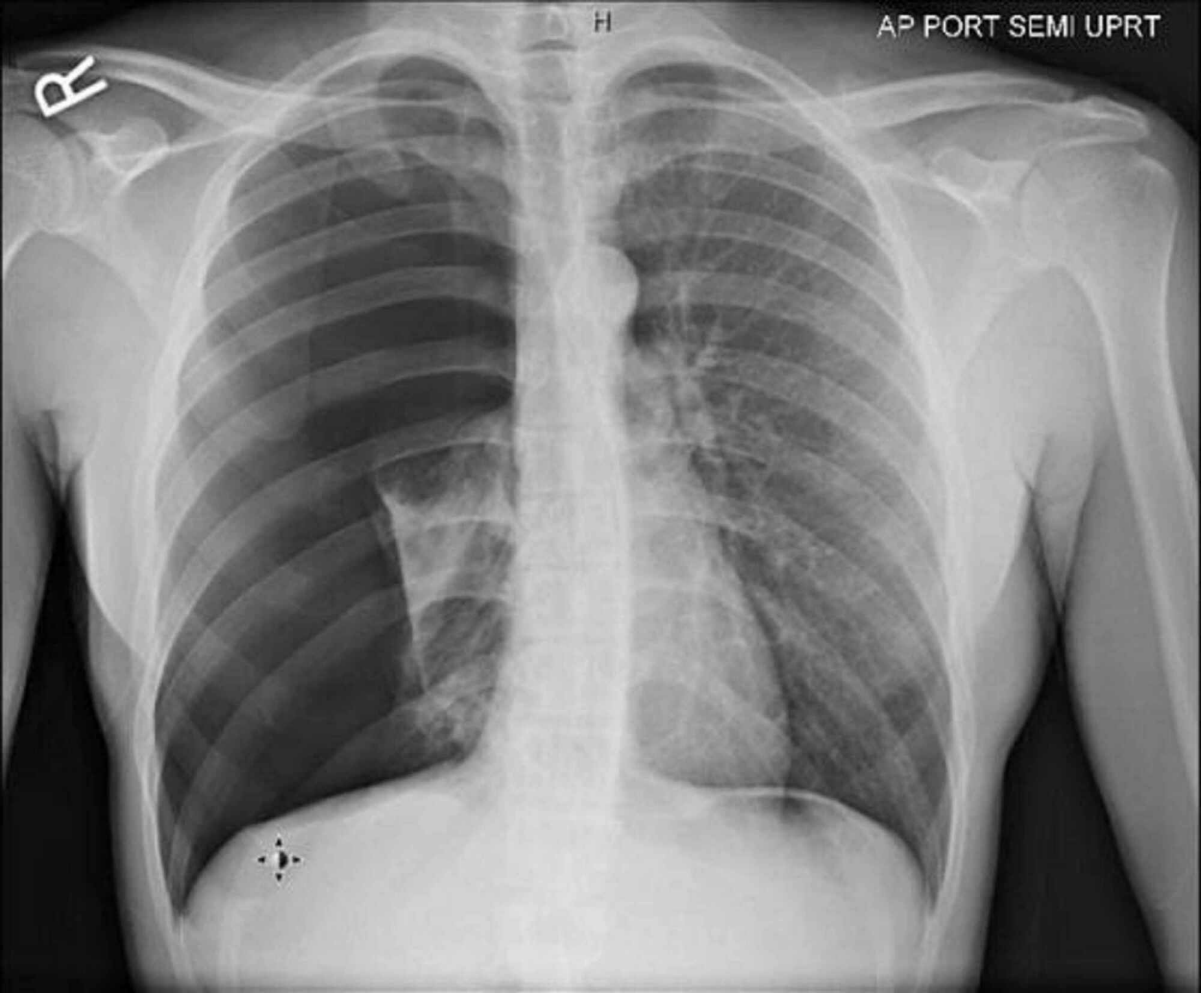
Chest x-ray (CXR) shows large right-sided pneumothorax with a complete collapse of the right lung.

Complete blood count (CBC), basic metabolic panel, and alpha-1 antitrypsin level were within the normal range. Chest x-ray (CXR) confirmed the diagnosis of a large right-sided pneumothorax with a complete collapse of the right lung (Figure 1).

**Figure 2:**
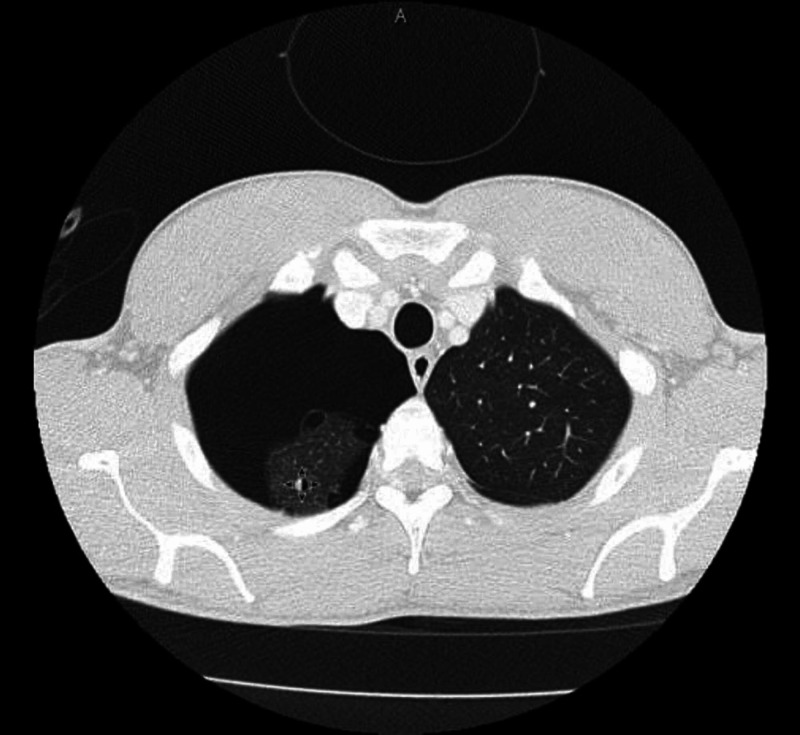
CT-thorax shows large right-sided pneumothorax with several small pleural blebs at the right apex and margin of the right upper lobe.

A chest tube was emergently placed upon the first presentation of pneumothorax, and continuous bubbling of the chest drain was noticed. Follow up computed tomography (CT) of thorax after 24 hours of chest tube placement revealed the persistence of large right-sided pneumothorax with several small pleural blebs at the right apex and margin of the right upper lobe (Figure 2).

**Figure 3:**
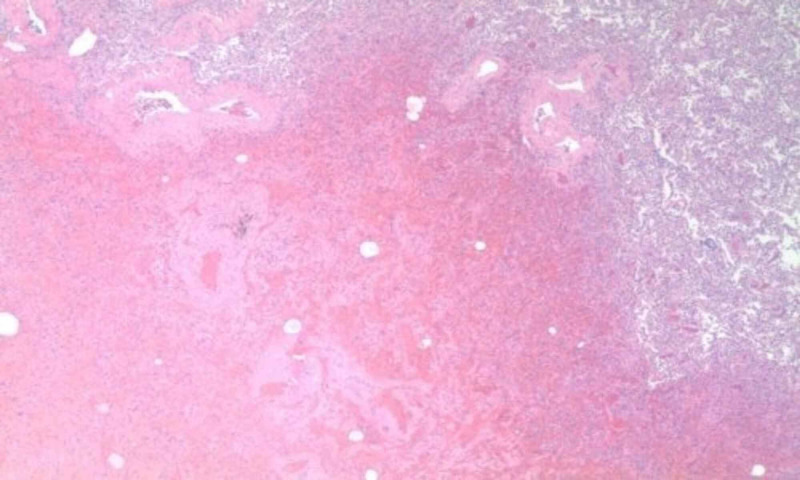
Histopathology: intraparenchymal hemorrhage and chronic inflammation.

Due to the persistence of pneumothorax and incomplete expansion of the lung despite chest drainage and suction, cardiothoracic surgeon performed video-assisted thoracoscopic surgery with right-upper and middle-lobe wedge resection of bullae, followed by pleurodesis. Pathology of resected segments revealed edema and intraparenchymal hemorrhage along with hyperplastic reactive changes and chronic inflammation (Figure 3).

The patient improved clinically and was discharged from the hospital after confirming re-expansion of the lung by serial CXRs. The patient was educated about the risk of smoking marijuana on his body and the importance of terminating the use of the drug. Unfortunately, the patient continued to smoke marijuana heavily, and he presented to the emergency room five months later with similar complaints of chest pain and shortness of breath. CXR confirmed recurrent right-sided pneumothorax for which a chest tube was placed. Serial post-drain CXRs showed re-expansion of the lung, and surgery was not required. The patient's clinical condition improved, and he was advised again before discharge about the need to stop using marijuana. 

## Discussion

Increasing evidence supports the association of marijuana smoking with bullous emphysema and spontaneous pneumothorax. A prospective study supports the correlation between marijuana abuse and primary spontaneous pneumothorax [10]. A case-controlled study by Stefani et al. revealed that cannabis smokers are at significant risk of developing bullous lung disease and have a higher incidence of tension pneumothorax compared to both tobacco smokers and nonsmokers [9]. 

Although the mechanism for bullae formation is still uncertain, the significantly larger puff volume and increased depth and time of inhalation with marijuana compared with tobacco smoking are suggested contributing mechanical factors [11]. Another precipitating factor is increased intra-alveolar pressure through coughing while breath-holding that may lead to shearing force on alveoli causing pneumothorax.

A study on mice by Helyes et al., designed to evaluate the chronic effects of daily marijuana inhalation on lungs, demonstrated histopathological changes similar to our patients’ lung parenchymal changes described in the pathology report. The study provides experimental evidence that marijuana causes inflammation and emphysema [12].

Literature review of bullous lung disease and spontaneous pneumothorax associated with marijuana abuse showed three similar case reports, as shown in Table 1.

**Table 1:** Case reports of spontaneous pneumothorax associated with marijuana abuse.

Author	Case Description
Mishra et al. [13]	30-year-old male with a 5-year history of marijuana smoking, no tobacco smoking, had right-sided pneumothorax with few apical bullae in the right lung.
Goodyear et al. [14]	23-year-old male with a 10-year history of marijuana smoking, no tobacco smoking, had bilateral spontaneous pneumothorax.
Gao et al. [15]	23-year-old male with a history of marijuana smoking for several years through a bong, no tobacco smoking, had bilateral large upper lobe bullae and recurrent pneumothorax.

Common etiologies for primary and secondary spontaneous pneumothorax were excluded in our patient as he had average height and weight and no past medical or surgical conditions. He did not have any family history of pneumothorax, and he denied using tobacco or any other recreational or illicit drugs. The patient did not have any connective tissue diseases based on the history and physical examination, like rheumatoid arthritis, ankylosing spondylitis, or systemic sclerosis. CT-thorax revealed several small pleural blebs at the right apex and margin of the right upper lobe, but there were no other radiological findings associated with secondary spontaneous pneumothorax, such as interstitial lung disease or bronchiectasis. All this considered, heavy marijuana abuse was thought to cause bullous lung disease leading to recurrent spontaneous pneumothorax in our patient.

## Conclusions

This case highlights the potential risks of marijuana abuse to lung health in the absence of tobacco use. Among young individuals presenting with emphysema, marijuana use should be considered in the differential diagnosis. We should keep in mind that marijuana abuse is associated with spontaneous pneumothorax, which can be recurrent. Lastly, physicians should take a detailed history of marijuana use and explain its harmful effects to patients.
